# Telehealth Perceptions Among US Immigrant Patients at an Academic Internal Medicine Practice: Cross-sectional Study

**DOI:** 10.2196/36069

**Published:** 2022-08-10

**Authors:** Susan Levine, Richa Gupta, Kenda Alkwatli, Allaa Almoushref, Saira Cherian, Dominique Feterman Jimenez, Greishka Nicole Cordero Baez, Angela Hart, Clara Weinstock

**Affiliations:** 1 UConn Health University of Connecticut Farmington, CT United States

**Keywords:** telemedicine, immigrant patient, immigrant, ethnic minority, cultural minority, patient satisfaction, telehealth, satisfaction, health care, health equity, digital health, patient experience

## Abstract

**Background:**

The use of telemedicine has increased dramatically through the COVID-19 pandemic. Although data are available about patient satisfaction with telemedicine, little is known about immigrant patients’ experience.

**Objective:**

We sought to investigate patients’ experiences with telehealth compared to in- person visits between immigrants and nonimmigrants. We wanted to identify and describe next visit preferences within the Farmington University of Connecticut Internal Medicine practice to ultimately guide suggestions for more equitable use and accessibility of visit options.

**Methods:**

A total of 270 patients including 122 immigrants and 148 nonimmigrants were seen by 4 Internal Medicine providers in an in-person (n=132) or telemedicine (n=138) university practice setting. Patients were queried between February and April 2021, using an adaptation of a previously validated patient satisfaction survey that contained standard questions developed by the Consumer Assessment of Healthcare Providers and Systems Program. Patients seen via in-person visits completed a paper copy of the survey. The same survey was administered by a follow-up phone call for telemedicine visits. Patients surveyed spoke English, Spanish, or Arabic and were surveyed in their preferred language. For televisits, the same survey was read to the patient by a certified translator. The survey consisted of 10 questions on a Likert scale of 1-5. Of them, 9 questions assessed patient satisfaction under the categories of access to care, interpersonal interaction, and quality of care. An additional question asked patients to describe and explain the reasons behind next visit preferences. Survey question responses were compared by paired t tests.

**Results:**

Across both immigrant and nonimmigrant patient populations, satisfaction with perceived quality of care was high, regardless of visit type (*P*=.80, *P*=.60 for televisits and *P*=.76, *P*=.37 for in-person visits). During televisits, immigrants were more likely to feel providers spent sufficient time with them (*P*<.001). Different perceptions were noted among nonimmigrant patients. Nonimmigrants tended to perceive more provider time during in-person visits (*P*=.006). When asked to comment on reasons behind next televisit preference, nonimmigrant patients prioritized convenience, whereas immigrants noted not having to navigate office logistics. For those who chose in-person visits, both groups prioritized the need for a physical exam.

**Conclusions:**

Although satisfaction was high for both telemedicine and in-person visits across immigrant and nonimmigrant populations, significant differences in patient priorities were identified. Immigrants found televisits desirable because they felt they spent more time with providers and were able to avoid additional office logistics that are often challenging barriers for non-English speakers. This suggests opportunities to use information technology to provide cultural and language-appropriate information throughout immigrants’ in-person and telemedicine visit experience. A focus on diminishing these barriers will help reduce health care inequities among immigrant patients.

## Introduction

Telemedicine, defined as the remote diagnosis and treatment of patients by means of telecommunication technology, is an aspect of the broader entity telehealth. Telehealth refers to the delivery and facilitation of health and health-related services via digital communication technologies [1]. Methods to assess telehealth and telemedicine quality include measures of patient experience as well as patient satisfaction. Patient experience includes the range of interactions patients have with the health care system, including the care they receive, access to information, and communication. Patient satisfaction, on the other hand, is a narrower term defined as how well a patient’s expectations about a health encounter are met [2].

Telemedicine has been well studied and shown to enhance access to care in remote populations [3-6]. In rural settings, telemedicine is associated with decreased travel costs and increased access to social support [7]. Studies of video visits in subspecialty care have demonstrated the benefits of convenience and increased accessibility to cardiovascular, wound, and home care [8-14]. The use of telemedicine is increasing due to these benefits; but most of our knowledge of patient satisfaction and experience is gleaned from disease-specific, subspecialist, or rural settings. The impact of telemedicine on primary care is less studied.

A study of MinuteClinic consumers reported high satisfaction with telemedicine visits, naming convenience and high quality as drivers of satisfaction [15]. Studies of patients’ perceptions of telemedicine in the primary care setting have shown mixed results. One study assessed video visits with primary care physicians immediately followed by in-person visits with the same provider; patients found video visits were less desirable [16]. Another study of interviews of patients following video visits with primary care providers suggested patients were quite satisfied with video visits, prioritizing convenience and privacy when assessing visit type [17].

Beginning in 2020, the use of telemedicine expanded so patients could safely seek health care during the COVID-19 pandemic. Concerns persist that despite these efforts to increase access, telemedicine may increase the health care equity divide [18]. The increased use of telemedicine for health care delivery has dropped from the initial surge but continues at higher than prepandemic levels, primarily in urban, higher-income, and White populations [19]. The increased number of patients using telemedicine, whether it is their preferred method or the only option available, serves as an opportunity to investigate patient satisfaction and equitable use of telemedicine. Previous research suggests most patients and clinicians report no difference in the overall quality of a video visit as compared to an office visit. Patients rate televisits highly and find them desirable due to saved travel time and costs [20].

There are not many studies on whether the quality of telehealth care patients receive is impacted by additional challenges of navigating cultural and language differences present in the telemedicine setting. A Canadian study found that in health care interactions, immigrant patients are often concerned about communication barriers due to cultural and language differences [21]. Previous work looking at attitudes toward telemedicine by minorities in the United States found that Latinos and African Americans were satisfied with the increased access and reduced wait time provided by telemedicine but had concerns about confidentiality, privacy, and physical absence of the provider [22]. Data from the 2011-2015 National Health Interview Survey assessed the use of eHealth services including making appointments and filling prescriptions via the internet as well as using patient portals among US natives, naturalized citizens, and noncitizens. Researchers found that naturalized citizens and noncitizens were less likely than US natives to access eHealth. The underutilization of eHealth among immigrants was linked to English proficiency [23].

Patient satisfaction is traditionally linked to access to care, improved interpersonal interactions, and perceived quality of care [20]. Whether these outcomes apply equally to telemedicine visits across immigrant and nonimmigrant populations is unclear. Given that the University of Connecticut Farmington Internal Medicine practice serves a large immigrant population, we were interested in investigating patient experiences with telehealth compared to in-person visits across immigrant and nonimmigrant patient populations. We sought to identify and explain the reasons behind immigrant visit preferences to ultimately guide recommendations to encourage equitable use and accessibility of visit options. A cross-sectional study design was used to allow researchers to compare populations in real time and quickly assess the acceptance of a relatively novel telemedicine service.

## Methods

### Study Setting

The Farmington Internal Medicine practice of UConn Health serves a 72% Medicare and Medicaid population, many of whom are non-US natives. Patients were seen from February to April of 2021 by 4 participating internists.

### Intervention

In March of 2020, in response to the COVID-19 pandemic, UConn Health deployed telemedicine to all outpatient practices. At the time of this study, telephone, video, and in-person visits were offered to all patients simultaneously based on staff screening criteria. Criteria for video visits included mild COVID-19 complaints not requiring inpatient evaluation or a chief complaint that the provider agreed could be adequately addressed by a limited patient-facilitated exam. Telephone visits were used primarily for complaints that providers felt did not require an exam. Telemedicine visits were conducted via video and telephone. Video visits were conducted via Zoom embedded in Epic. Medical assistants scheduled televisits, and a call center scheduled in-person visits. Medical assistants provided detailed telephone instructions in patient’s preferred language about how to join the televisit.

### Participant Recruitment and Patient Characteristics

Survey data were collected for both in-person and telemedicine visits. Participants were recruited if scheduled with the participating providers and if they spoke English, Spanish, or Arabic. Visits were deemed by providers to be appropriate for in-person visits versus televisits based on patient’s preference and the chief complaint.

### Survey Development

Patient experience [24] was assessed through the administration of a survey (Multimedia Appendix 1), using some dimensions adapted from an instrument developed by the Consumer Assessment of Healthcare Providers and Systems consortium [25] and validated in a study [20]. Patients were asked to describe their experience with their present telemedicine or in-person visit by rating their agreement on statements about access to care, interpersonal interaction, and quality of care as 1 (definitely agree), 2 (disagree), 3 (neutral), 4 (somewhat agree), and 5 (definitely agree). An additional question asked patients to describe and explain the reason behind their next visit preferences. Patients were asked to explain if their choice was based on convenience, time off from work, time with the provider, visit quality, or another reason.

### Survey Administration

During the study period, all patients seen by participating providers were offered participation in the study. For in-person visits, medical assistants obtained verbal consent and passed a paper copy of the survey to patients in their preferred language at the beginning of the visit. Patients then completed the survey. For televisits, the investigating medical students made a follow-up call, using a university language line interpreter. After obtaining verbal consent, the same survey was read to telemedicine participants in their preferred language.

### Statistical Analysis

Results include quantitative and descriptive subgroup comparisons. For survey questions on patient satisfaction (questions 1-9), 2-tailed paired *t* tests (at *P*<.05 significance) were calculated in Microsoft Excel and used to compare numbers of individual Likert scale question responses. Although opinions differ on how to best analyze Likert data, consensus exists that parametric tests are appropriate [26]. The satisfaction survey responses did not follow a normal distribution; therefore, percentages of individual responses rather than means were compared. For the question on next visit preference (question 10), some patients left the question blank, others provided some combination of multiple selections and write-in responses. Therefore, this question was analyzed without any formal statistics.

### Ethical Considerations

For Spanish or Arabic surveys, a translation to the appropriate language by a native speaker and then a back translation to English by a separate native speaker was performed to ensure accurate translation. Surveys were deidentified and blinded to providers. The study was approved by the University of Connecticut Institutional Review Board (21X-132-1), and translation protocols were followed.

## Results

Survey data were collected from 138 televisits and 132 in-person visits. These responses came from 122 immigrant and 148 nonimmigrant patients (Table 1 and 2).

**Table 1:** Patient characteristics (N=270).

Characteristics			Participants^a^, n (%)		Televisits (n=138), n (%)		In-person visits (n=132), n (%)
**Female immigrant^b^**							
	Total	72 (51.1)		39 (28.3)		33 (25)	
	Aged 18-29	24 (17)		15 (10.9)		9 (6.8)	
	Aged 30-35	47 (33.3)		24 (17.4)		23 (17.4)	
	Aged >56	1 (0.7)		0 (0)		1 (0.8)	
**Female, US born**							
	Total	69 (48.9)		37 (26.8)		32 (24.2)	
	Aged 18-29	39 (27.6)		18 (13)		21 (15.9)	
	Aged 30-35	26 (17.4)		15 (10.9)		11 (8.3)	
	Aged >56	4 (2.7)		4 (2.9)		0 (0)	
**Male immigrant**							
	Total	50 (38.7)		24 (17.4)		26 (19.7)	
	Aged 18-29	21 (16.3)		10 (7.2)		11 (8.3)	
	Aged 30-35	29 (22.5)		14 (10.1)		15 (11.4)	
	Aged >56	0 (0)		0 (0)		0 (0)	
**Male, US born**							
	Total	79 (61.2)		38 (27.5)		41 (31.1)	
	Aged 18-29	46 (35.6)		24 (17.4)		22 (16.7)	
	Aged 30-35	30 (23.3)		14 (10.1)		16 (12.1)	
	Aged >56	3 (2.3)		0 (0)		3 (2.3)	

^a^Total female participants: n=141, 52.2%; total male participants: n=129, 47.8%.

^b^Born outside of the United States.

**Table 2:** Participant characteristics based on the country of origin (N=270; in-person visits: n=132, 51%; televisits: n=138, 49%).

Country of birth and preferred language^a^		In-person visit (n=132), n (%)	Televisit^b^ (n=138), n (%)
**Argentina**			
	Spanish	1 (0.7)	4 (1.5)
**Colombia**			
	English	2 (1.4)	2 (1.5)
	Spanish	0 (0)	10 (7.6)
**Costa Rica**			
	Spanish	4 (2.9)	2 (0.8)
**Dominican Republic**			
	Spanish	2 (1.4)	2 (1.5)
**Ecuador**			
	Spanish	3 (2.2)	4 (3.0)
**El Salvador**			
	Spanish	2 (1.4)	3 (1.5)
**Guatemala**			
	Spanish	3 (2.2)	2 (1.5)
	English	3 (2.2)	0 (0)
**Peru**			
	Spanish	1 (2.9)	3 (2.3)
	English	1 (0.7)	6 (4.5)
**Venezuela**			
	Spanish	4 (2.9)	3 (1.5)
**Afghanistan**			
	Arabic	4 (2.9)	3 (1.5)
**Iran**			
	Arabic	0 (2.2)	2 (1.5)
	English	3 (2.2)	0 (0)
**Jordan**			
	Arabic	3 (2.2)	2 (1.5)
**Morocco**			
	Arabic	3 (2.2)	1 (0.8)
**Pakistan**			
	Arabic	4 (2.9)	4 (3.0)
	English	4 (2.9)	2 (1.5)
**Qatar**			
	Arabic	0 (0)	1 (0.8)
**Somalia**			
	Arabic	2 (1.4)	1 (0.8)
**Syria**			
	Arabic	6 (4.3)	5 (3.8)
**Turkey**			
	Arabic	2 (1.4)	3 (2.3)
**United States (nonimmigrant)**			
	English	75 (54.3)	73 (55.3)

^a^Patients were surveyed in their preferred language.

^b^Of the visits, 63 were via video and 75 via telephone.

### Patient Experiences With Telemedicine And In-Person Visits

Survey response rates for in-person visits and televisits were 89% and 78%, respectively. During televisits, immigrants were more likely than nonimmigrants to feel providers spent enough time with them (*P*<.001), whereas nonimmigrants felt providers spent more time with them during in-person visits (*P*=.006). There were no significant differences between immigrant and nonimmigrant perceptions of quality of care between visit types (*P*=.80 and *P*=.60 for televisits; *P*=.76 and *P*=.37 for in-person visits). All but 2 patients preferred next visits to be congruent visit types (Tables 3 and 4).

**Table 3:** Summary of responses by those who participated in telemedicine visits (n=138), characterized by immigrant (n=63) and nonimmigrant (n=75) experiences.

Survey domains, items, and patient type				Definitely disagree, n (%)		Disagree, n (%)		Neutral, n (%)		Somewhat agree, n (%)		Definitely agree, n (%)		*P* value^a^	
**Access to care**															
	I was able to schedule today’s visit soon enough														.21
		Immigrant	0 (0)		0 (0)		1 (2)		7 (11)		55 (87)				
		Nonimmigrant	0 (0)		0 (0)		2 (2)		14 (19)		59 (79)				
	I saw the provider I wanted to see today														.007
		Immigrant	0 (0)		0 (0)		2 (3)		3 (5)		58 (92)				
		Nonimmigrant	0 (0)		0 (0)		12 (16)		6 (8)		57 (76)				
	I got the type of visit I wanted today														.003
		Immigrant	0 (0)		0 (0)		1 (2)		3 (5)		59 (93)				
		Nonimmigrant	0 (0)		0 (0)		6 (8)		14 (19)		55 (73)				
**Interpersonal interaction**															
	My provider spent enough time with me														<.001
		Immigrant	0 (0)		0 (0)		0 (0)		4 (6)		55 (94)				
		Nonimmigrant	0 (0)		0 (0)		4 (5)		24 (32)		47 (63)				
	My provider listened to me														.09
		Immigrant	0 (0)		0 (0)		0 (0)		3 (5)		60 (95)				
		Nonimmigrant	0 (0)		0 (0)		0 (0)		10 (13)		65 (87)				
	My provider addressed all my concerns														.53
		Immigrant	0 (0)		0 (0)		0 (0)		3 (5)		60 (95)				
		Nonimmigrant	0 (0)		0 (0)		2 (3)		2 (3)		71 (94)				
**Quality of care**															
	My provider showed me respect														.74
		Immigrant	0 (0)		0 (0)		0 (0)		3 (5)		60 (95)				
		Nonimmigrant	0 (0)		0 (0)		2 (3)		2 (3)		71 (94)				
	The quality of care was excellent														.80
		Immigrant	0 (0)		0 (0)		1 (2)		4 (6)		58 (92)				
		Nonimmigrant	0 (0)		0 (0)		2 (3)		2 (3)		71 (94)				
	I would recommend the provider I saw today to my family														.60
		Immigrant	0 (0)		0 (0)		0 (0)		2 (4)		61 (96)				
		Nonimmigrant	0 (0)		0 (0)		1 (1)		2 (3)		72 (96)				
**Next visit preference**															
	(If today was a televisit) I prefer a televisit for my next visit														.07
		Immigrant	0 (0)		0 (0)		0 (0)		0 (0)		63 (100)				
		Nonimmigrant	2 (3)		0 (0)		0 (0)		0 (0)		73 (97)				

^a^Paired *t* test comparing responses between immigrants and nonimmigrants.

**Table 4:** Summary of responses by those who participated in in-person visits (n=132), characterized by immigrant (n=59) and nonimmigrant (n=73) experiences.

Survey domains, items, and patient type				Definitely disagree, n (%)		Disagree, n (%)		Neutral, n (%)		Somewhat agree, n (%)		Definitely agree, n (%)		*P* value^a^	
Access to care															
	I was able to schedule today’s visit soon enough														.83
		Immigrant	0 (0)		1 (2)		1 (2)		5 (8)		52 (88)				
		Nonimmigrant	0 (0)		2 (3)		2 (3)		4 (5)		65 (89)				
	I saw the provider I wanted to see today														.20
		Immigrant	0 (0)		0 (0)		0 (0)		0 (0)		59 (100)				
		Nonimmigrant	0 (0)		0 (0)		0 (0)		2 (3)		71 (97)				
	I got the type of visit I wanted today														.27
		Immigrant	0 (0)		0 (0)		0 (0)		1 (2)		58 (98)				
		Nonimmigrant	0 (0)		0 (0)		0 (0)		0 (0)		73 (100)				
Interpersonal interaction															
	My provider spent enough time with me														.006
		Immigrant	0 (0)		0 (0)		0 (0)		1 (2)		39 (66)				
		Nonimmigrant	1 (1)		0 (0)		0 (0)		0 (0)		69 (95)				
	My provider listened to me														.11
		Immigrant	0 (0)		0 (0)		0 (0)		4 (7)		55 (93)				
		Nonimmigrant	0 (0)		0 (0)		0 (0)		1 (1)		72 (99)				
	My provider addressed all my concerns														.15
		Immigrant	0 (0)		0 (0)		0 (0)		5 (8)		54 (92)				
		Nonimmigrant	0 (0)		0 (0)		0 (0)		2 (3)		71 (97)				
Quality of care															
	My provider showed me respect														.37
		Immigrant	0 (0)		0 (0)		0 (0)		0 (0)		59 (100)				
		Nonimmigrant	0 (0)		0 (0)		0 (0)		0 (0)		73 (100)				
	The quality of care was excellent														.76
		Immigrant	0 (0)		0 (0)		0 (0)		1 (2)		58 (98)				
		Nonimmigrant	0 (0)		0 (0)		1 (3)		0 (0)		72 (99)				
	I would recommend the provider I saw today to my family														.37
		Immigrant	0 (0)		0 (0)		0 (0)		2 (4)		59 (100)				
		Nonimmigrant	1 (1)		0 (0)		1 (1)		2 (3)		72 (99)				
Next visit preference															
	(If today was a televisit) I prefer a televisit for my next visit														.37
		Immigrant	0 (0)		0 (0)		0 (0)		0 (0)		59 (100)				
		Nonimmigrant	0 (0)		0 (0)		0 (0)		0 (0)		73 (100)				

^a^Paired *t* test comparing responses between immigrants and nonimmigrants.

### Reasons Behind Next Visit Preference

**Figure 1:**
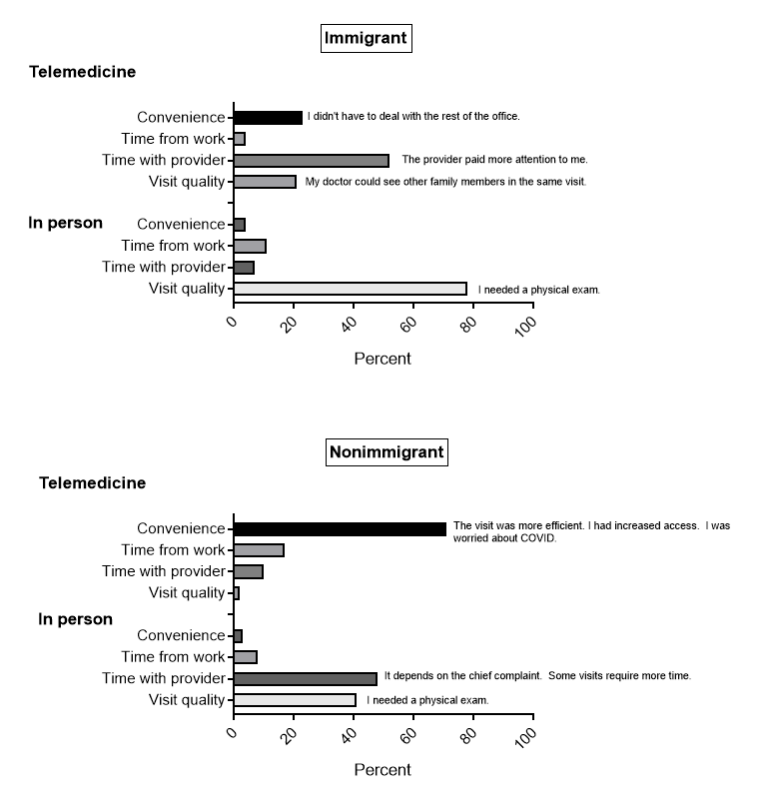
Reasons behind next visit preferences.

When asked to describe and explain the reason for preferring a telemedicine or in-person visit, nonimmigrants prioritized convenience when choosing televisits. Convenience was explained as a more efficient visit as well as an option to obtain a timelier appointment. Immigrants, on the other hand, prioritized time with the provider when preferring telemedicine visits, explained as the advantage of not having to navigate the rest of the office. Common reasons across patient groups for preferring in-person visits included visit quality, explained as the perceived need for a detailed physical exam (Figure 1).

## Discussion

### Principal Findings

We sought to investigate patient experience and describe and explain the reasons behind telehealth visit preferences compared to in-person visits between immigrants and nonimmigrants. We hoped to use these preferences to guide suggestions for a more equitable use and accessibility of visit options. In terms of rating the patient experience of access to care, both patient groups felt that they were able to schedule a soon enough appointment regardless of the visit type (televisits: *P=*.21; in-person visits: *P*=.83). Immigrants were more likely to feel they saw the provider they wanted in a televisit, whereas both patient groups felt they had access to the provider they wanted in an in-person visit (televisits: *P*=.007; in-person visits: *P*=.20). In assessing access to the preferred visit type, immigrants were more likely to feel they got the type of visit they originally wanted when the visit was a televisit. When the visit was an in-person visit, both patient groups felt they received the visit type they wanted (televisit *P=*.003; in-person visit: *P*=.27). Immigrants were more likely to feel providers spent enough time with them when the visit was a televisit (*P*<.001), whereas nonimmigrants felt they had more time with their providers during an in-person visit (*P*=.006). This difference did not seem to depend on other interpersonal cross-cultural communication factors. There seemed to be no differences perceived in listening (televisits: *P*=.09; in-person visits: *P*=.11*)*, showing respect (televisits: *P*=.74; in-person visits: *P*=.15), or addressing concerns (televisits: *P=*.53; in-person visit: *P*=.37) across immigrant and nonimmigrant patient populations. An explanation for immigrants’ perception of additional time with providers during televisits included not having to navigate additional office logistics. Nonimmigrants seemed to have different priorities in visit preferences. They seemed to prioritize convenience in telemedicine visits described as less time off from work and increased efficiency and access. Nonimmigrants also prioritized time with providers but for seemingly different reasons, including when the need for a physical exam or a more complex chief complaint arose.

### Comparison With Previous Studies

Previous studies have described favorable patient experience with video visits when applied to disease-specific conditions in rural settings [27-29]. One study found disease-specific video visits in rural settings favorable because they were associated with decreased travel costs, less time off from work, and a greater ability to tailor care to patient and family needs [7]. These findings are consistent with the nonimmigrant subset of our population who found telemedicine visits favorable primarily for the reasons of convenience and less time off from work. Although telemedicine visits have been used at increasing rates in primary care settings, studies are largely limited to nonimmigrant patient populations. In nonimmigrant populations, a variety of methods have been used to assess patient experience with telemedicine, including patient surveys and interviews [24]. A mixed survey and interview study [30] reported increased satisfaction with telemedicine based on convenience and the ability to access care safely during the pandemic. Telehealth was felt by patients to be most appropriate for routine follow-ups when a physical exam was not necessary, especially when there was already an established patient-provider relationship [30]. The fact that all our patients were seen by their primary providers may have increased their acceptance of televisits. Both immigrant and nonimmigrant populations prioritized the need for a physical exam in evaluating in-person visits. The fact that immigrants found televisits desirable seems to somewhat conflict with previous work that has shown immigrants are less likely to access eHealth services including scheduling appointments via the internet and using electronic patient portals [23]. However, these tasks may be inherently more complex to navigate for a non-US native than having a telemedicine appointment. The fact that our medical assistants, with the aid of a translator, walked immigrants through the process of joining a televisit likely increased immigrants’ access to these appointments and their acceptance of them. As telemedicine visits were typically scheduled by medical assistants rather than our call center, medical assistants may have been protective of patients whom they knew had difficulty navigating the system, and they likely pushed a little more to get immigrant patients in with their primary providers. Our immigrants’ acceptance of telemedicine may also be due in part to having a more tech-savvy immigrant population, as many were refugees and as such had recently successfully navigated incredibly complex logistics. Telehealth videoconferencing has been successfully used to coordinate care for immigrants with chronic conditions such as hepatitis C and latent tuberculosis. The advantage and acceptance of this visit type was linked to the ease of coordinating care between provider, patient, and subspecialist [31]. Similarly in our study, immigrants noted that an advantage of the telemedicine visit was the ability of the provider to see multiple family members simultaneously. Our study conflicts in part with previous work that shows Latinos question the absence of providers in televisits [22]. Investigators point to a concern about privacy and digital access for Latinos based on income, insurance, and documentation status. Our patients were insured, documented, and may have had more digital access.

### Study Limitations

A limitation of the generalizability of this study is the inability to segregate data by English proficiency. The immigrant population in this study included primarily newly arrived immigrants. Although the countries of origin represented in this study are numerous, participant numbers from individual countries are small. The survey used in the study was validated in English and administered according to the Institutional Review Board’s back translation protocols. However, given the small numbers of individual countries represented, it is impossible to draw culturally specific conclusions. Finally, detailed patient interviews might have more fully uncovered reasons behind patient preferences.

### Conclusions

Although nonimmigrants preferred televisits because of their convenience, immigrant patients preferred televisits due to the perceived time spent with providers. This preference was found in the absence of any perceived differences in other interpersonal communication factors and supported by additional write-in responses suggesting a possible reason for this preference, namely that the telemedicine environment seems to eliminate some of the inherent barriers found in navigating the office. This study suggests that multiple opportunities exist to use information technology to provide cultural and language-appropriate information throughout immigrants' health care experience.

As we continue to expand telemedicine, it is important to understand the different priorities and unique barriers experienced by immigrant populations. Although university practices often have access to robust telephone translation services, these services are less accessible outside of the in-person visit encounter. Resources within the electronic medical record to communicate in other languages could be developed and applied to additional aspects of the patient experience, including visit scheduling, appointment reminders, portal use, patient instructions, and telephone reminders. Patient navigators and a wider array of language options in web-based instructions might also be used to help ensure scheduling and follow-ups. Provider education to optimize telemedicine examination techniques and support alternative scheduling models may expand provider uptake [32]. Advocacy for broader reimbursement of telephone visits might also improve immigrant access to telemedicine when video visits are financially less accessible.

## Appendix

Multimedia Appendix 1 Patient experience survey.
