# Supplementary material for: Telehealth Perceptions Among US Immigrant Patients at an Academic Internal Medicine Practice: Cross-sectional Study
Source: JMIR Hum Factors. 2022 Aug 10;9(3):e36069. doi: 10.2196/36069 (PMC9403821; doi:10.2196/36069)
Supplement: Multimedia Appendix 1 [file humanfactors_v9i3e36069_app1.pdf]

Background: **(staff to complete)** **patient number: ---------**

**Seen by PCP today**	Yes	No	
**How long have you lived in the US (years)?**			
**Country of origin**			
**Preferred language**			
**Type of visit** (circle one)	tele = 1	video = 2	live = 3
**Reason for visit** (ECV or FOV) (circle one)	Specific indication =		

Satisfaction: **(patient to complete)**

(1=definitely disagree, 2=disagree, 3=neutral, 4=somewhat agree, 5=definitely agree)

**Access to care**	**1**	**2**	**3**	**4**	**5**
**1.** I was able to schedule today's visit soon enough					
**2.** I saw the provider I wanted to see today					
**3.** I got the type of visit (telemedicine vs live) I wanted today					
**Interpersonal interaction**					
**4.** In this visit, my provider spent enough time with me					
**5.** In this visit, my provider listened to me					
**6.** In this visit, my provider addressed all my concerns					
**7.** In this visit my provider showed me respect					
**Quality of care**					
**8.** In this visit, the quality of care was excellent					
**9.** I would recommend the provider I saw today to family					
**Next visit preference**					
**10.** (If today was a Televisit), I prefer a televisit for my next visit					
**11.** (If today was a Livevisit) I prefer a live visit for my next visit.					
**12.** Why or why not? (circle one)	Convenience	time lost from work	time with provider	Visit quality	Other (use back)
